# Improved fat signal suppression for coronary MRA at 3T using a water-selective adiabatic T_2_-Prep technique

**DOI:** 10.1186/1532-429X-15-S1-O5

**Published:** 2013-01-30

**Authors:** Andrew J Coristine, Ruud B van Heeswijk, Matthias Stuber

**Affiliations:** 1 CardioVascular Magnetic Resonance (CVMR), Centre d'Imagerie BioMédicale (CIBM), Lausanne, Switzerland; 2 Faculté de Biologie et de Médecine, Université de Lausanne, Lausanne, Switzerland

## Background

In MRI, fat signal suppression is mandatory for unambiguous visualization of cardiac anatomy but remains challenging at high field. Fat saturation usually directly precedes the imaging portion of a cardiac MRI sequence. It is often used in conjunction with other magnetization preparation modules, such as T_2_ Preparation (or T_2_-Prep), to improve blood/myocardium contrast. We propose a water-selective, adiabatic T_2_-Prep that combines T_2_-weighting with an intrinsic fat signal attenuation. Preliminary in vivo results are discussed.

## Methods

The first radiofrequency (RF) pulse of a +90°,180°,180°,-90° adiabatic T_2_-Prep was reduced in bandwidth, from 1250 Hz to 285 Hz. Water is thus excited but off-resonant fat magnetization is left longitudinal. Meanwhile, the bandwidth of the final RF pulse (-90°) remains large (1250 Hz), encompassing both water and fat frequencies. It thus restores the magnetization of T_2_-prepared water, while fat magnetization is tipped down and then spoiled. The RF excitation angles of the first and last pulse were experimentally increased to ±120°, to further reduce fat signal via inversion recovery. To demonstrate how these modifications can easily combine with other fat saturation strategies, images were acquired with and without conventional frequency selective fat saturation (CHESS), for both the water-selective adiabatic T_2_-Prep and the unmodified adiabatic T_2_-Prep. Volume targeted 3D imaging of the right coronary artery was performed for all 4 sequences in 6 healthy adults. All images were acquired on a 3T Siemens TRIO using a navigator- and cardiac-gated segmented k-space Cartesian gradient echo sequence, with FoV 360x258 mm, matrix size 240x216, 1.5 mm slice thickness, 15 k-space lines/heartbeat, TE T2-Prep = 40 ms, RF excitation angle 15°, TE/TR/T_Acq_=2.37/5.37/80.55 ms. Images were reformatted and analyzed using semi-automated vessel tracking software (Soap-Bubble). Fat suppression efficacy was compared using vessel sharpness measurements and signal-to-noise ratio (SNR) quantification in selected regions (abdominal fat, epicardial fat, blood, myocardium).

## Results

**Figure 1:**
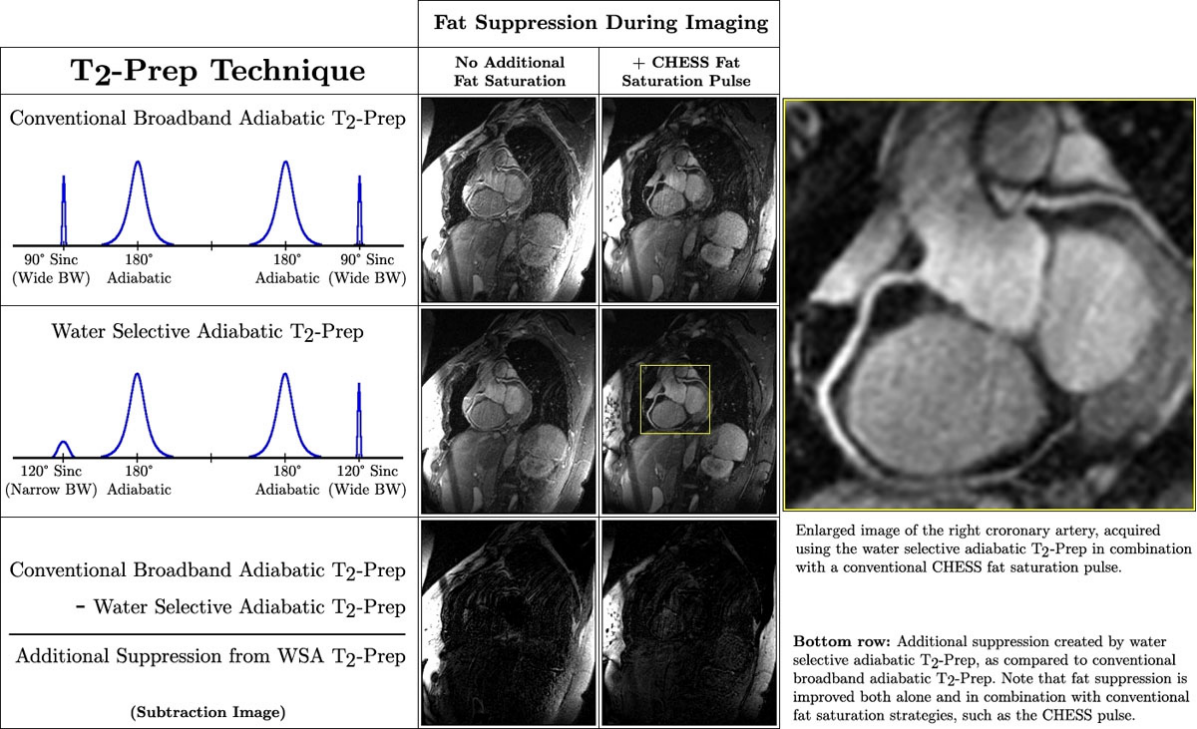
Increased fat signal suppression of the variable bandwidth (BW) water selective adiabatic (WSA) T_2_-Prep technique as compared to conventional broadband adiabatic T_2_-Prep. Fat suppression is improved both with and without an additional frequency-selective fat saturation (CHESS) pulse.

Sample images are shown in Figure 1, with corresponding SNR measurements in Table 1. When no complementary fat saturation was used, the water-selective adiabatic T_2_-Prep reduced abdominal and epicardial fat signals by 36% and 16% (p<0.001 and p<0.05) and improved vessel sharpness from 43.8% to 47.3% (p<0.05), as compared to the unmodified T_2_-Prep. When a CHESS pulse was added prior to imaging, the water-selective adiabatic T_2_-Prep reduced abdominal and epicardial fat signals by a further 54% and 30% (p<0.002 and p<0.02) and improved vessel sharpness from 68.4% to 72.7% (p<0.05), as compared to unmodified T_2_-Prep + CHESS. Blood and myocardium SNRs were not significantly affected.

**Table 1:** Mean SNR (± one S.D.) for conventional broadband adiabatic T_2_-Prep vs. water selective adiabatic T_2_-Prep, both with or without an additional frequency-selective fat saturation (CHESS) pulse.

	No Additional Fat Saturation		+ CHESS Fat Saturation Pulse	
Region of Interest	Conventional Adiabatic T_2_-Prep	Water Selective Adiabatic T_2_-Prep	Conventional Adiabatic T_2_-Prep	Water Selective Adiabatic T_2_-Prep

Abdominal Fat	339.3 (±76.1)	217.9 (±73.1) *	165.5 (±25.6)	75.6 (±31.2) *

Epicardial Fat	122.0 (±48.8)	102.2 (±51.5) *	28.6 (±7.4)	20.0 (±2.6) *

Blood	67.3 (±5.4)	79.1 (±25.2)	77.2 (±11.0)	78.2 (±19.7)

Myocardium	39.0 (±1.2)	47.3 (±13.5)	40.8 (±4.2)	45.7 (±10.8)

* indicates a significant difference (p<0.05 or smaller).

## Conclusions

A water-selective adiabatic T_2_ Preparation module significantly improves fat saturation in 3T coronary MRA and should be considered as a potential addition to conventional fat saturation strategies.

## Funding

N/A

